# Fatty Acid Composition of Developing Sea Buckthorn (*Hippophae rhamnoides* L.) Berry and the Transcriptome of the Mature Seed

**DOI:** 10.1371/journal.pone.0034099

**Published:** 2012-04-27

**Authors:** Tahira Fatima, Crystal L. Snyder, William R. Schroeder, Dustin Cram, Raju Datla, David Wishart, Randall J. Weselake, Priti Krishna

**Affiliations:** 1 Department of Biology, University of Western Ontario, London, Ontario, Canada; 2 Department of Agricultural, Food & Nutritional Science, University of Alberta, Edmonton, Alberta, Canada; 3 Agroforestry Development Centre, Agriculture and Agri-Food Canada, Indian Head, Saskatchewan, Canada; 4 Plant Biotechnology Institute, National Research Council, Saskatoon, Saskatchewan, Canada; 5 Departments of Computing Science and Biological Sciences, University of Alberta, Edmonton, Alberta, Canada; Max Planck Institute for Chemical Ecology, Germany

## Abstract

**Background:**

Sea buckthorn (*Hippophae rhamnoides* L.) is a hardy, fruit-producing plant known historically for its medicinal and nutraceutical properties. The most recognized product of sea buckthorn is its fruit oil, composed of seed oil that is rich in essential fatty acids, linoleic (18∶2ω-6) and α-linolenic (18∶3ω-3) acids, and pulp oil that contains high levels of monounsaturated palmitoleic acid (16∶1ω-7). Sea buckthorn is fast gaining popularity as a source of functional food and nutraceuticals, but currently has few genomic resources; therefore, we explored the fatty acid composition of Canadian-grown cultivars (ssp. *mongolica*) and the sea buckthorn seed transcriptome using the 454 GS FLX sequencing technology.

**Results:**

GC-MS profiling of fatty acids in seeds and pulp of berries indicated that the seed oil contained linoleic and α-linolenic acids at 33–36% and 30–36%, respectively, while the pulp oil contained palmitoleic acid at 32–42%. 454 sequencing of sea buckthorn cDNA collections from mature seeds yielded 500,392 sequence reads, which identified 89,141 putative unigenes represented by 37,482 contigs and 51,659 singletons. Functional annotation by Gene Ontology and computational prediction of metabolic pathways indicated that primary metabolism (protein>nucleic acid>carbohydrate>lipid) and fatty acid and lipid biosynthesis pathways were highly represented categories. Sea buckthorn sequences related to fatty acid biosynthesis genes in Arabidopsis were identified, and a subset of these was examined for transcript expression at four developing stages of the berry.

**Conclusion:**

This study provides the first comprehensive genomic resources represented by expressed sequences for sea buckthorn, and demonstrates that the seed oil of Canadian-grown sea buckthorn cultivars contains high levels of linoleic acid and α-linolenic acid in a close to 1∶1 ratio, which is beneficial for human health. These data provide the foundation for further studies on sea buckthorn oil, the enzymes involved in its biosynthesis, and the genes involved in the general hardiness of sea buckthorn against environmental conditions.

## Introduction

Sea buckthorn (*Hippophae rhamnoides* L.), belonging to the family Elaeagnaceae, is a winter-hardy, deciduous shrub indigenous to Asia and Europe. The female plants produce yellow to orange fruit that is highly valued for its unique nutritional and medicinal properties. Sea buckthorn berries, together with leaves and bark, have been used for hundreds of years in Russia and China for medicinal and nutritional purposes [1]. Furthermore, as a beautiful, hardy (temperature, salt and drought resistant), nitrogen-fixing plant that rapidly develops an extensive root system, sea buckthorn is also used as an ornamental for enhancing wildlife habitat, and for preventing soil erosion and conserving essential nutrients.

Published literature indicates that sea buckthorn berries are highly enriched in vitamins (C, A, E and K), organic acids, amino acids, fatty acids and antioxidants [carotenoids (lycopene, *β-*carotenes, zeaxanthin) and flavonoids (quercetin, kaempferol, isorhamnetin, myricetin)] [2]–[5]. The pulp and seed oils have unique compositions and contain bioactive compounds, including phytosterols, carotenoids, and tocopherols. The pulp oil has a high content of palmitoleic acid (16∶1*^cis^* ^Δ9^) (up to 43%). Since this fatty acid is a major constituent of skin fat, the pulp oil is used for cosmetic and healing purposes [6]. The seed oil contains linoleic acid (18∶2*^cis^* ^Δ9,12^) (up to 42%) and α-linolenic acid (18∶3*^cis^* ^Δ9,12,15^) (up to 39%) in high amounts [7] and in close to 1∶1 ratio, which is different from the polyunsaturated fatty acid composition of major vegetable oils [8]. Numerous health benefits, including antiatherogenic, cardioprotective, antiplatelet and antiulcer activities of the seed oil, as well as antioxidative activity of leaf extracts, have been demonstrated using cell culture and animal models [9]–[12]. Sea buckthorn berries are also rich in polyphenols, which function as antioxidants [13]–[15]. Sea buckthorn flavones have been linked with inhibition of thrombosis and hypertension [16], [17], and promotion of wound healing [18]. The positive effects of berries and its extracts have also been demonstrated in human subjects [19], [20] thus, both historical accounts and scientific research indicate that this plant has immense nutritional and medicinal potential.

In the last decade, interest in sea buckthorn’s health benefits has increased considerably in many countries. Although several plantations have come up in Canada, there is limited information on the metabolite composition of the currently grown cultivars. Furthermore, this plant is currently an unexplored source of novel traits and genes. So far, only about 2787 high quality sequences (>100 bp in length) derived from a cDNA library prepared from leaf tissue have been submitted to the EST database (dbEST) of NCBI [21]. A *glycerol-3-phosphate acyltransferase* (*GPAT*) gene was cloned and its expression was shown to increase in cold-stressed leaves of sea buckthorn [22]. This is the extent of gene sequence information that is available in this plant species.

Considering the repertoire of bioactives and unusual fatty acid composition of sea buckthorn berry, and its potential as a health product, we analysed the seed and pulp fatty acid compositions in four Canadian-grown cultivars and generated the first sea buckthorn seed transcriptome using high-throughput 454 sequencing methodology to uncover genes related to oil biosynthesis and other important metabolic pathways as well as stress response pathways. From the 89,141 putative unigenes in our 454 dataset, we identified most of the genes involved in fatty acid biosynthesis. The current dataset forms the first comprehensive genomic resource for sea buckthorn, which establishes a basis for dissecting metabolic pathways related to the formation of oil and bioactive components, and developing strategies to enhance the production of desirable compounds.

## Results and Discussion

### Fatty Acid Composition of Whole Berry, Seed and Pulp Oil

**Figure 1:**
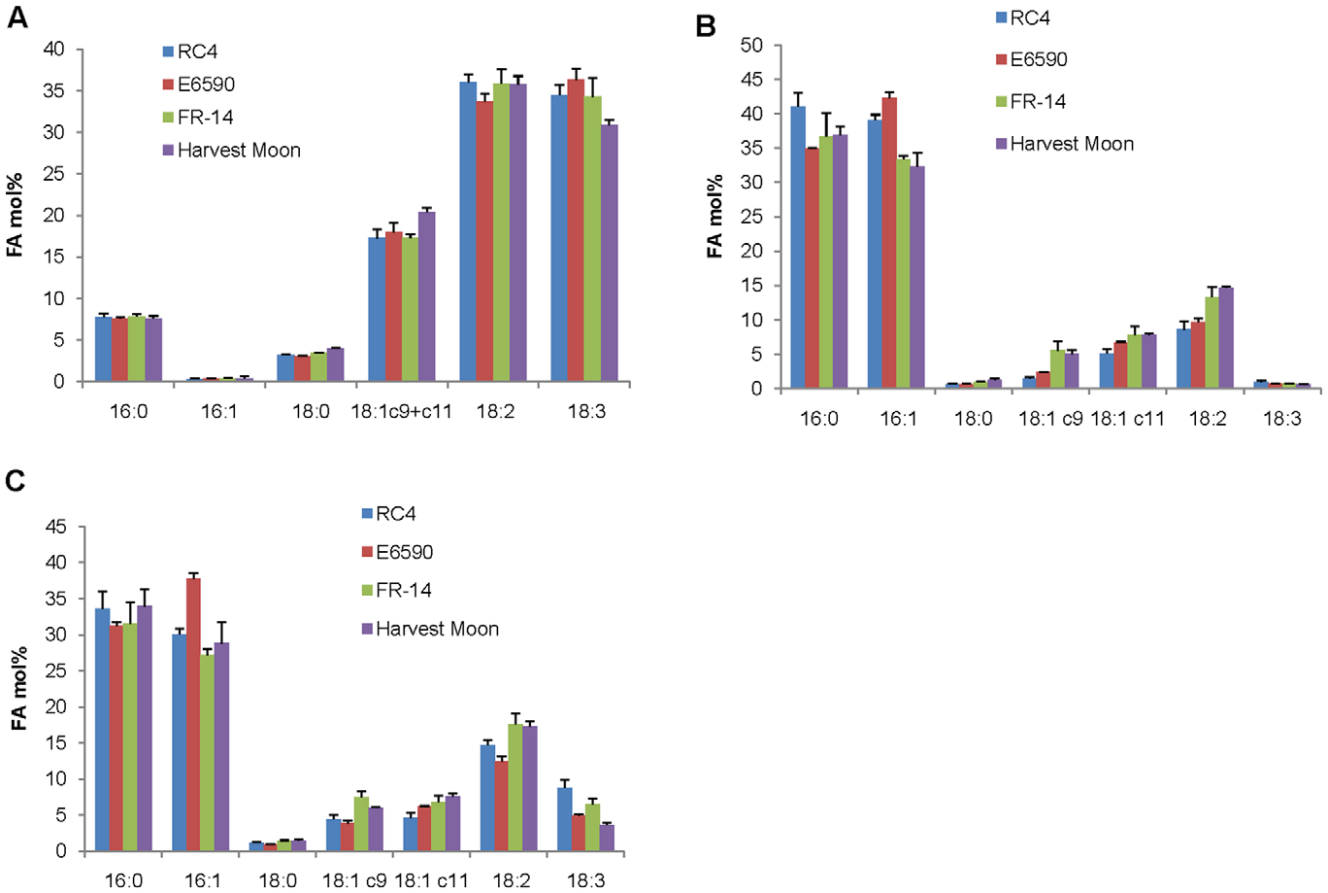
Fatty acid composition of total lipids in A) seed oil, B) pulp oil and C) whole berry oil of four cultivars RC-4, E6590, FR-14 and Harvest Moon. Results represent the mean ± SD of three biological replicates. Minor fatty acids (accounting in total for <3% of the fatty acid composition) are not shown. 16∶0, palmitic acid; 16∶1, palmitoleic acid; 18∶0, stearic acid; 18∶1c9, oleic acid; 18∶1c11, *cis*-vaccenic acid; 18∶2, linoleic acid; 18∶3, α-linolenic acid.

Fully ripe fruit of four superior sea buckthorn cultivars (RC-4, E6590, Harvest Moon and FR-14), selected from a seedling population of *H. rhamnoides* ssp. *mongolica* and grown in Saskatchewan, Canada, were harvested and flash frozen. RC-4 is very hardy, and together with FR-14, has a desirable crown form, while Harvest Moon and E6590 have other superior agronomic traits such as fruit mass and yield, long pedicel, absence of thorns and easy harvest. Fatty acids were analysed in total lipid extracts of whole berries, pulp and seeds by gas chromatography-mass spectrometry (GC-MS). In seed oil, linoleic acid and α-linolenic acid accounted for roughly equal proportions of the total composition, at 33–36% and 30–36%, respectively. Oleic acid (18∶1*^cis^* ^Δ99^) and its isomer (18∶1*^cis^* ^Δ11^) were present at a combined content of 17–20%, followed by palmitic acid (16∶0) at ∼7% (Figure 1A). Palmitoleic acid and stearic acid (18∶0) were each present at <4% of the total fatty acids. There was little variation among the four cultivars in the fatty acid compositions of the seed oils. The four cultivars were derived from ssp. *mongolica*, but only RC-4 and FR-14 have a similar genetic background while Harvest Moon and E6590 have different genetic backgrounds. The lack of variation in the fatty acid composition of the four cultivars used suggests that either the fatty acid compositions do not vary within subspecies or that the growing environment is a major factor controlling fatty acid composition.

In the pulp oil, the dominant fatty acid was the monounsaturated palmitoleic acid at 32–42%, followed by palmitic acid at 34–41% (Figure 1B). Interestingly, two isomers of 18∶1 were detected, 18∶1*^cis^* ^Δ9^ at 1–5% and 18∶1*^cis^* ^Δ11^ (*cis*-vaccenic acid) at 5–7%. Linoleic acid accounted for 8–14% of the total composition, and all other fatty acids, including α-linolenic and stearic acids were each at <2% of the total fatty acids. The results represented in Figure 1A and B confirm in the Canadian-grown cultivars what can be considered as the trademark of sea buckthorn oil - the generally high levels of α-linolenic acid and linoleic acid in seed oil, and of palmitoleic acid in pulp oil. The contents of various fatty acids in the whole berry oil were palmitic acid at 31–33%, palmitoleic acid at 28–37%, stearic acid at <2%, 18∶1 (*cis*Δ9 and *cis*Δ11) each at 3–8%, linoleic acid at 12–18%, and α-linolenic at 3–8% (Figure 1C). Due to the abundance of pulp tissue over seed, the whole berry oil composition is more reflective of the pulp oil composition.

**Figure 2:**
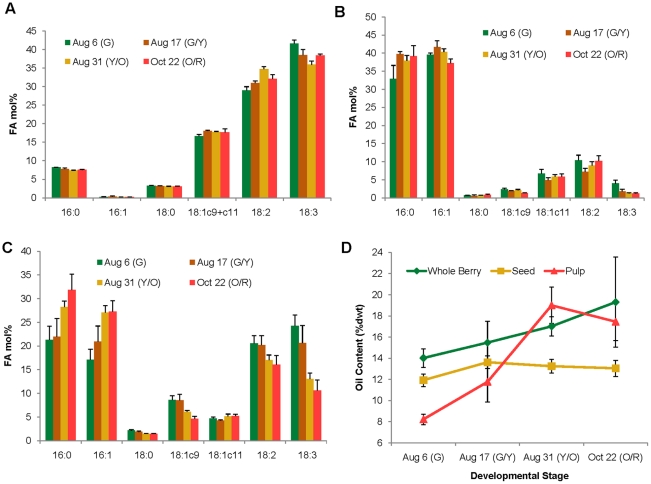
Fatty acid composition of total lipids during berry development stages of RC-4 cultivar. The oil samples were extracted from A) seed, B) pulp and C) whole berries of RC-4 cultivar harvested at different stages. Results represent the mean ± SD of three biological replicates. Minor fatty acids (accounting in total for <5% of the total fatty acid composition) are not shown. D) The accumulation of oil in whole berries, seeds and pulp over four developmental stages described by fruit color: G: green, G/Y: green/yellow, Y/O: yellow/orange, O/R: orange/red. 16∶0, palmitic acid; 16∶1, palmitoleic acid; 18∶0, stearic acid; 18∶1c9, oleic acid; 18∶1c11, *cis*-vaccenic acid; 18∶2, linoleic acid; 18∶3, α-linolenic acid.

The accumulation of major fatty acids during fruit development was examined in seed, pulp and whole berry at four different stages of development of RC-4. Berries were collected, using color as a marker, when the fruits were green (G), green/yellow (G/Y), yellow/orange (Y/O) and orange/red (O/R) (Figure S1). The overall fatty acid profiles of various stages of fruit development roughly mirrored that of mature seeds, but there was a decrease in unsaturated fatty acids, especially α-linolenic acid, in seed and whole berry oils, over the course of fruit development (Figure 2A, C). In pulp oil, palmitic acid increased after the green stage and then stayed relatively constant over the remaining developmental stages (Figure 2B), while both palmitic and palmitoleic acid continued to increase in whole berry at later stages (Figure 2C), likely reflecting the increase in pulp mass and oil content relative to the contribution from the seeds (Figure 2D). The oil content of the seeds was already relatively high at the green stage, and stayed relatively constant following the green/yellow stage, while the oil content in pulp and whole berry continued to increase (Figure 2D). This, along with the relatively large contribution of pulp oil to whole berry fatty acid composition in the green stage, suggests that oil deposition begins very early in fruit development and the green stage sampled in this study does not represent the earliest stage of oil biosynthesis.

Interestingly, oil-based bioactive compounds, such as α-tocopherol and β-sitosterol, were reported as being highest in sea buckthorn fruit fraction at an ‘early maturity stage’ [23]. Although we cannot exactly correlate the ‘early maturity stage’ with the developmental stages of berries used in the current study, from an oil-related nutritional standpoint it would appear that unripened sea buckthorn berries are superior to fully ripened berries. This information is important for deciding the optimum harvest time of sea buckthorn berries in order to fully harness the health benefits of the oil. The health benefit of sea buckthorn seed oil lies in the low ω-6∶ω-3 ratio (1∶1), in part due to the anti-inflammatory effects of ω-3 fatty acids [24]. Currently, the Western diet contains a very high (15∶1) ω-6∶ω-3 fatty acid ratio, which has been linked with cancer, cardiovascular, inflammatory and autoimmune diseases [25], [26]. α-Linolenic acid is a biosynthetic precursor of long chain ω-3 polyunsaturated fatty acids, eicosapentaenoic acid (EPA; 20∶5*^cis^* ^Δ5,8,11,14,17^) and docoasahexaenoic acid (DHA; 22∶6*^cis^* ^Δ4,7,10,13,16,19^), which are nutritionally important fatty acids abundant in fish oil. With growing concerns regarding accumulation of environmental pollutants in fish oil as well as sustainability of marine fish stocks, plants rich in ω-3 fatty acids may offer a sustainable source of these beneficial fatty acids. The fatty acid profile of sea buckthorn seed is particularly interesting since it contains a high proportion of ω-3 fatty acids relative to ω-6 fatty acids. With the exception of flaxseed oil, which contains >50% α-linolenic acid, such high levels of ω-3 fatty acids are uncommon in seed oils, and are usually accompanied by a high ω-6∶ω-3 ratio. Considerable research efforts are being put towards the production of ω-3 fatty acid-enriched products, both for human consumption and use as animal feed [27]–[29]. Sea buckthorn seed oil, with its high α-linolenic levels together with a near 1∶1 ratio of ω-6∶ω-3 fatty acids represents a very balanced source of polyunsaturated fatty acids for human health and nutrition.

High levels of palmitoleic acid are present in only few plants, such as sea buckthorn pulp oil and macadamia nut oil. Interest in this fatty acid arises from its lower susceptibility to oxidation compared to polyunsaturated fatty acids, which may confer functional advantages such as stability during frying and baking. To date, limited information is available on the effects of palmitoleic acid on cardiovascular risk factors. A recent study focusing on the effects of dietary palmitoleic acid in hamsters, however, found no adverse effects on plasma lipoprotein profiles or aortic cholesterol accumulation [30], while another study found palmitoleic acid to have effects similar to palmitic acid (saturated fatty acid) on plasma total and low density lipoprotein-cholesterol concentrations [31]. At present, the best application of palmitoleic acid is in cosmetic manufacturing, but there is enormous potential as sustainable feedstock for producing industrially important octane, which is used as a comonomer in the expanding production of linear low-density polyethylene [32]. To demonstrate proof of concept that ω-7 fatty acid levels can be increased to high levels in plants, Arabidopsis was metabolically engineered to produce ω-7 fatty acid to as much as 71% [32]. Since sea buckthorn is low-input, high yielding plant that can grow on marginal lands, it could serve as a sustainable plant feedstock for industrially important chemicals due to its unusually high palmitoleic acid level. Also, if the sea buckthorn pulp Δ9 desaturase enzyme is found to have higher specificity for conversion of 16∶0 to 16∶1Δ9 than its orthologs from other species, the gene could be used in increasing monounsaturated fatty acid levels in plants and other organisms.

### Sequencing and Assembly of Reads Generated Using 454 GS FLX Platform

**Figure 3:**
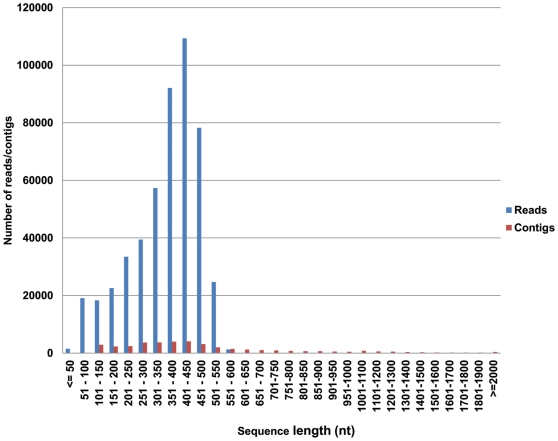
Size distribution of sea buckthorn 454 reads and assembled contigs.

Seeds were isolated from O/R stage berries of the RC-4 cultivar. Total RNA was extracted from these seeds and used for non-normalized ds-cDNA library construction. 454 sequencing of the seed cDNA collections provided a total of 500,392 reads, which were assembled using two sequence data management (SDM) and analysis platforms, Fiesta 2 and GenomeQuest, to produce a non-redundant dataset for functional annotation and comparative analysis. This allowed analysis of the 454 data in different ways and produced datasets that reinforced each other. The sequences were first clustered by TIGR Gene Indices clustering tools (TGICL) [33] and archived on Fiesta 2 annotation platform at the Plant Biotechnology Institute (PBI), Saskatoon. The 500,392 reads were clustered and assembled into 37,482 contigs and 51,659 singletons, resulting in 89,141 putative unigenes (Table 1). The second method of clustering and annotation was done on the GenomeQuest SDM platform in two steps, dependant on the ability of the assembler software Newbler from Roche to only process 300,000 reads at one time. First, the 500,392 reads were split into two buckets of 250,196 sequences each in FASTA for assembly. Using this method, 497,182 non-redundant reads were assembled into 39,330 contigs, 58,062 singletons, and 97,392 unigenes (Table 1). The size distribution of reads and unigenes is shown in Figure 3. The most prominent size range for the reads and unigenes was 401–450 nts, and the average length and GC content of the unigenes was 474 nts and 37.6%, respectively.

**Table 1:** Summary of sea buckthorn sequences obtained on the 454 platform.

	Fiesta 2	GenomeQuest
Raw reads	500,392	500,392
Clustered/Non-redundant reads	500,392	497,182
Contigs	37,482	39,330
Range of contigs length	51 – 6,630 nts	89 – 7,176 nts
Singletons	51,659	58,062
Range of singletons length	40 – 642 nts	50 – 627 nts
Unigenes	89,141	97,392

**Figure 4:**
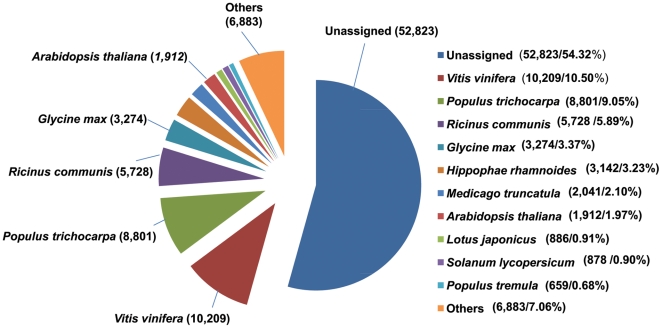
Species distribution of top BLAST hits of sea buckthorn sequences with other plant species.

Next, the 97,392 unigenes obtained using the GenomeQuest SDM platform were annotated with their best hits in public databases by running Rapid Annotation Process (RAP) against PLN (plant, fungal, and algal sequences) in GenBank to find distribution of sea buckthorn sequences against other organism sequences. RAP is based on nucleotide sequence-similarity searching algorithm. Using this method, 44,413 unigenes (45.68%) had hits to 760 organisms, while 52,823 unigenes (54.32%) remained unassigned (Figure 4). Maximum hits were obtained with sequences from *Vitis vinifera* (10,209), followed by *Populus trichocarpa* (8801), *Ricinus communis* (5728), *Glycine max* (3274) and others. The top-hit species distribution (grape, populus, and oil plants such as castor and soybean) may reflect the fruit, shrub/tree and oilseed characteristics of sea buckthorn, respectively. The unassigned sequences likely result from sequences being too short to find high matches, regulatory RNA sequences, 5′ and 3′ untranslated regions of transcripts, sequencing artifacts or novel gene sequences. Some of these sequences are likely to be of biological relevance and will be explored in the future.

The 89,141 unigenes in Fiesta 2 were searched against both Arabidopsis and plant databases in UnipProt using BLASTX with an e-value cutoff of 1e-6 to find their homologues. A total of 41,166 (46%) and 43,494 (49%) unigenes had significant hits with sequences in TAIR and UnipProt Plants, respectively.

### Gene Ontology Annotation of Sea Buckthorn Unigenes

We used MetaCyc (MetaCyc.org) [34] as a reference database with the Pathway Tools software to computationally predict the metabolic network of sea buckthorn seed. The total number of pathways identified was 239, which corresponded to 10,011 sequences. Within the category “biosynthesis”, the number of pathways in decreasing order was: amino acid, fatty acid and lipid, secondary metabolite, and carbohydrate biosynthesis (Table 2). Thus, the metabolic pathway prediction accurately reflects biosynthesis and storage of proteins, lipids, and carbohydrates as primary processes in seeds. In addition to the primary metabolites, seeds also store a diverse range of secondary metabolites, including flavonoids, phytosterols, saponins and other compounds of medicinal value. Some of these compounds are involved in defense against pathogens and predators, while others influence seed maturation and dormancy [35]. Since sea buckthorn is regarded as a medicinal plant rich in secondary metabolites, sequences related to secondary metabolite biosynthesis should be of particular interest.

**Table 2:** Biosynthesis pathways in sea buckthorn seed transcriptome based on MetaCyc pathway collections.

Pathway	Number
Amines and polyamines biosynthesis	7
Amino acids biosynthesis	43
Aminoacyl-tRNA charging	1
Aromatic compounds biosynthesis	1
Carbohydrates biosynthesis	23
Cell structures biosynthesis	3
Co-factors, prosthetic Groups, electron carriers biosynthesis	23
Fatty acids and lipids biosynthesis	34
Hormones biosynthesis	2
Metabolic regulators biosynthesis	2
Nucleosides and nucleotides biosynthesis	4
Secondary metabolites biosynthesis	26

**Figure 5:**
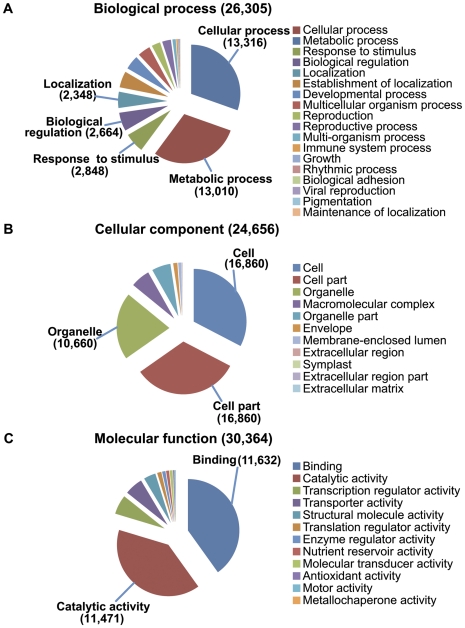
Gene Ontology annotation of sea buckthorn unigenes. A) Biological process; B) Cellular component; C) Molecular function.

The Gene Ontology (GO) system was used to summarize possible functional classifications of the unigenes via assignment of Arabidopsis gene identifiers with the strongest BLASTX alignments to the corresponding sea buckthorn sequences. Of the 89,141 sequences, 33,705 (37.8%) could be annotated under the three major GO categories: “biological process” (Figure 5A), “cellular component” (Figure 5B) and “molecular function” (Figure 5C). Within the category “biological process” (26,305 unigenes), the two highly represented GO terms were “cellular process” (50.6%) and “metabolic process” (49.5%), followed by “response to stimulus” (10.8%) and other categories (Figure 5A). Due to our interest in oil biosynthesis, we followed the GO term “metabolic processes” (Figure S2) with subterms in this order: cellular metabolic process > cellular lipid metabolic process > lipid biosynthetic process > fatty acid biosynthetic process/glycerolipid biosynthetic process/others. The distribution of sequences under the subterm, “lipid biosynthetic process”, is shown in Table S1. We found a preponderance of sequences related to jasmonic acid and wax biosynthesis genes within the GO category “fatty acid biosynthetic process”, and to *diacylglycerol O-acyltransferase 1* (*DGAT1*) and *phospholipid/glycerol acyltransferase 9* (*GPAT9*) within the GO category “glycerolipid biosynthetic process” (Table S2). A search for other fatty acid biosynthesis genes within the 434 sequences shown in Table S1 identified sequences related to *acetyl-CoA carboxylase* (*ACCase*), *3-ketoacyl-ACP-synthase III* (*KAS III*), *3-oxoacyl-ACP reductase* (*KAR*), *3-hydroxyacyl-ACP dehydratase* (*HAD*), *Δ12 fatty acid desaturase* (*FAD2*), *stearoyl-ACP desaturase* (*SAD*) and *acyl-ACP thioesterases* (*FATA, FATB*) (Table S2).

### Identification of Sea Buckthorn Genes Involved in Lipid Biosynthesis

**Figure 6:**
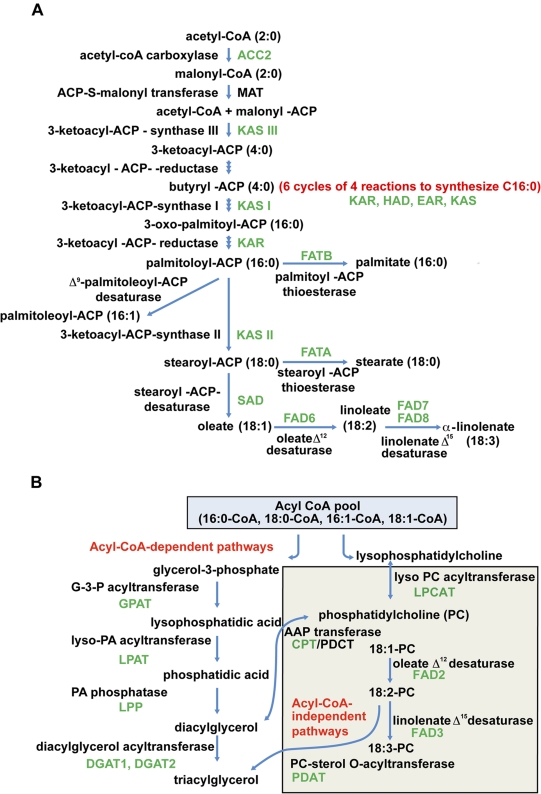
Sea buckthorn sequences associated with fatty acid A) and triacylglycerol B) biosynthetic pathways. Enzymes with names in full are represented on the left, and with names in short are represented on the right. Enzymes known to function at specific steps of the pathway are represented in black. Enzymes for which sea buckthorn sequences have been identified are shown in green. EAR, enoyl-acyl-ACP-reductase; HAD, 3-hydroxyacyl-ACP dehydratase. Enzymes responsible for phosphatidylcholine acyl editing in TAG assembly (LPCAT, CPT/PDCT, PDAT) are shown in the shaded box in figure 6B.

To identify all known lipid biosynthesis genes in sea buckthorn, the relevant Arabidopsis gene sequences were used to query (e-value cutoff of 1e-5) the sea buckthorn dataset in Fiesta 2. Enzymes for which gene sequences were identified in sea buckthorn are shown in green in Figure 6A,B. With the exception of *ACP-S-malonyl transferase* (*MAT*), sequences for most other enzymes involved in the biosynthesis and elongation of fatty acids were identified in the sea buckthorn seed transcriptome presented here. With the exception of *ACP-S-malonyl transferase* (*MAT*), sequences for most other enzymes involved in the biosynthesis and elongation of fatty acids were identified in the sea buckthorn seed transcriptome presented here.

The number of hits (reads, as well as contigs+singletons) obtained against each Arabidopsis fatty acid biosynthesis gene, and the nucleotide sequence identities between the Arabidopsis gene sequences and the most closely related sea buckthorn contigs are shown in Table 3. The large number of hits obtained for some genes likely represents several possibilities: different members of the gene family in sea buckthorn, different fragments of the same gene, or sequencing and assembly errors. The number of reads for any gene is dependent on the size and also on transcript abundance in the starting material. *SAD*, which catalyzes the desaturation of stearoyl-ACP to form oleoyl-ACP, had the largest number of reads at 364 (Table 3, Table S3), which is consistent with the relatively high levels of oleic acid and its downstream products in sea buckthorn seed oil. *DGAT1*, which catalyzes the final step in triacylglycerol (TAG) assembly, had the second highest number of reads at 142.

**Table 3:** List of enzymes and the corresponding Arabidopsis genes involved in fatty acid biosynthesis along with number of sea buckthorn ESTs, and nucleotide identities between Arabidopsis genes and closest sea buckthorn sequences.

Function	Enzyme Symbol	Enzyme description	Arabidopsis gene ID	Sea buckthorn contigs+singletons	Reads/gene	Sequences most closely related to Arabidopsis genes	Sequence length (nt)	Global gene identity (%)	Local gene identity (%)
**Initiation**	ACC2	Acetyl-CoA carboxylase	AT1G36180	9+7 = 16	66	CL1337Contig3	1801	18.5	71.5
**Elongation**	KAS III	3-Ketoacyl-ACP-synthase III	AT1G62640	1+4 = 5	8	CL10403Contig1	936	39.4	72.9
	KAS I	3-Ketoacyl-ACP-synthase I	AT5G46290	1+2 = 3	4	CL9108Contig1	551	24.0	57.2
	KAS II	3-Ketoacyl-acp-sunthase II	AT1G74960	1+1 = 2	5	CL9108Contig1	551	24.3	68.9
	HAD	3-hydroxyacyl-ACP ehydratase	AT2G22230	1+1 = 2	3	CL18597Contig1	416	37.7	39.9
	KAR	3-oxoacyl-ACP-reductase	AT1G24360	3+1 = 4	22	CL4678Contig2	972	60.7	68.1
	EAR	Enoyl-acyl-ACP-reductase	AT2G05990	0+2 = 2	2	FX9V3UK02IU9LS	430	25.6	43.8
**Desaturation**	SAD	Stearoyl-ACP desaturase	AT2G43710	15+3 = 18	364	CL1Contig5118	1768	43.6	62.0
	FAD2	Omega-6 fatty acid desaturase	AT3G12120	8+2 = 10	37	CL7573Contig1	836	29.9	66.8
	FAD3	Omega-3 fatty acid desaturase	AT2G29980	9+2 = 11	39	CL5598Contig1	1203	64.9	68.2
	FAD6	Omega-6 fatty acid desaturase	AT4G30950	5+2 = 7	27	CL12952Contig1	640	35.3	54.2
	FAD7	Fattyacid desaturase 7	AT3G11170	9+4 = 13	41	CL5560Contig1	1251	38.3	43.4
	FAD8	Fattyacid desaturase 8	AT5G05580	9+4 = 13	41	CL5560Contig1	1251	36.9	41.5
**Thioesterase**	FATA	Acyl-ACP thioesterase	AT3G25110	2+1 = 3	17	CL3499Contig1	1293	52.9	68.3
	FATB	Acyl-ACP thioesterases B	AT1G08510	4+3 = 7	43	CL4458Contig2	879	26.1	70.8
**TAG synthesis**	GPAT1	1-acylglycerol-3-phosphate O-acyltransferase	AT1G06520	0+2 = 2	2	FX9V3UK02JX8KP	423	15.1	43.8
	LPAT4	Lyso PA acyltransferase	AT1G75020	1+0 = 1	4	CL9684Contig1	536	31.1	40.2
	LPP1	Phosphatidate phosphatase 1	AT2G01180	2+1 = 3	27	CL2162Contig1	1341	47.6	57.9
	LPP2	Phosphatidate phosphatase 2	AT1G15080	2+1 = 3	27	CL2162Contig2	500	38.9	53.9
	**DGAT1**	Type 1 diacylglycerol acyltransferase	AT2G19450	16+4 = 20	142	CL71Contig14	2277	45.8	60.6
	**DGAT2**	Type 2 diacylglycerol acyltransferase	AT3G51520	4+0 = 4	11	CL12762Contig1	549	24.0	67.0
	LPCAT	Lyso PC acyltransferase	AT1G80950	3+0 = 3	30	CL1897Contig1	1944	42.1	67.1
	CPT	Choline phosphotransferase	AT1G13560	3+1 = 4	33	CL616Contig5	1078	52.9	76.3
	PDAT	PL diacylglycerol transferase	AT5G13640	7+2 = 9	40	CL4682Contig1	1532	39.3	43.1

In most oilseeds, lipids are stored primarily as TAGs, with the major constituents being 16- and 18-carbon saturated and unsaturated fatty acids. Figure 6A shows the pathway leading from acetyl-CoA to synthesis of nascent 16–18 carbon fatty acids in the plastid, and the pathways for TAG assembly in the endoplasmic reticulum are shown in Figure 6B. TAG can be synthesized through the sequential acyl-CoA dependent acylation of a glycerol backbone, or by various acyl-CoA independent routes which are believed to facilitate the incorporation of polyunsaturated fatty acids into TAG [36], [37]. Several lines of evidence indicate that the level of DGAT activity may have a substantial effect on the flow of carbon into seed oil. For example, in Arabidopsis, mutant alleles of *DGAT1* accumulate less TAG than the wild type [38], while overexpression of *DGAT1* increases oil content in transgenic seeds [39].

High expression of *SAD* and *ACCase* has also been associated with increased oil content in high-oil maize [40]. SAD catalyzes desaturation of stearoyl-ACP to form oleoyl-ACP. A thioesterase (FATA) catalyzes the release of the oleoyl moiety from ACP, and the resulting free fatty acid is exported from the plastid and is esterified to CoA. Another acyl-ACP thioesterase, FATB, has higher affinity for saturated acyl-ACPs such as palmitoyl-ACP and stearoyl-ACP [41]. The fatty acid desaturation (FAD) enzymes, FAD2 (Δ12 desaturase) and FAD3 (Δ15 desaturase), along with FAD6 and FAD7/FAD8, are responsible for sequential modification of oleic acid to linoleic acid and linolenic acid, thereby increasing the polyunsaturated to saturated (P:S) fatty acid ratios. Given the potential involvement of *ACC2, SAD, DGAT1, FAD2, FAD3, FAD6* and *FAD7/FAD8* in the accumulation of major fatty acids in mature sea buckthorn seeds (Figure 6A,B), these results warrant further studies related to expression, substrate specificity or regulation of activity of enzymes related to lipid biosynthesis. We have isolated full-length ORF cDNAs for *DGAT1, DGAT2, SAD* and *FAD2*; the encoded sea buckthorn proteins share amino acid identities of 65%, 62%, 84% and 70%, respectively, with the Arabidopsis orthologs.

### Transcript Profiling of Lipid Biosynthesis-related Genes During Berry Development

**Figure 7:**
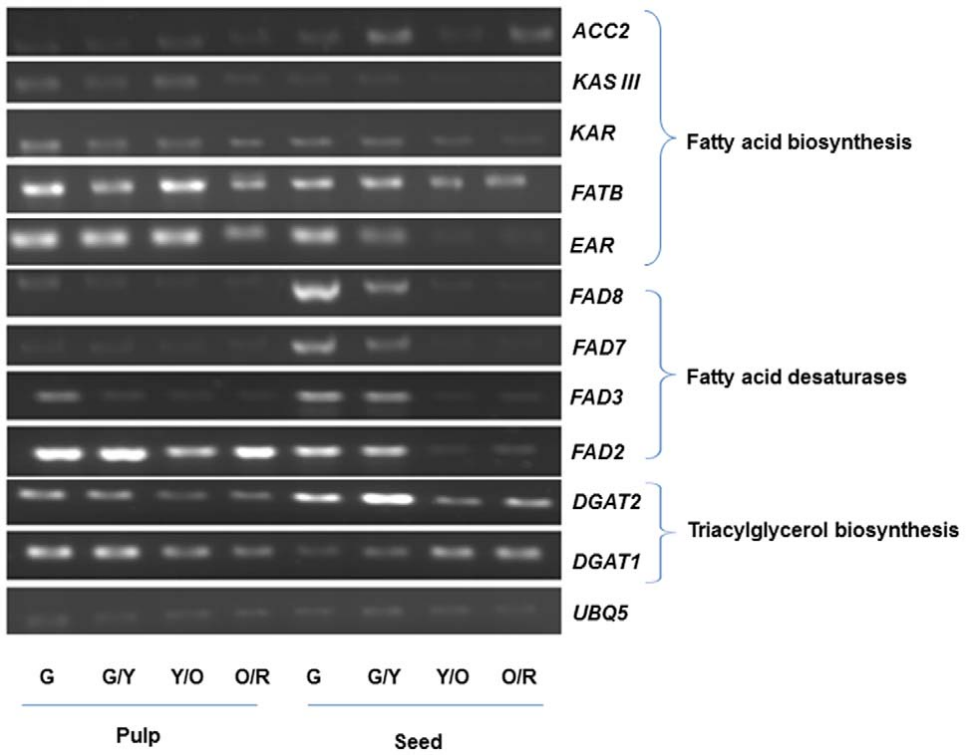
RT-PCR analysis of genes involved in fatty acid and triacylglycerol biosynthesis in seed and pulp tissues at different developmental stages of fruit from RC-4 cultivar. G, G/Y, Y/O and O/R represent fruits harvested at green (6 August), green/yellow (17 August), yellow/orange (31 August) and orange/red (22 October) stages, respectively. *Ubiquitin5* (*UBQ5)* was used as loading control.

To see if any correlations could be found between fatty acid compositions in developing fruits (Figure 2) and expression patterns of lipid biosynthesis genes, we analyzed transcript abundance by RT-PCR in RC-4 pulp and seeds at the four different developmental stages described in Figure S1. Most of the genes were expressed in both pulp and seeds, although the level of expression varied considerably between the tissue types and at different developmental stages (Figure 7). Genes involved in the early steps of fatty acid biosynthesis, *ACC2*, *KASIII* and *KAR*, exhibited relatively low transcript abundance throughout fruit development. Considering that oil accumulation was already well underway in the green stage (Figure 2D), this low-level expression of fatty acid biosynthesis genes may signify a shift toward desaturation and TAG assembly pathways. Indeed, expression of the desaturases, *FAD2, FAD3, FAD7* and *FAD8* peaked in the green or green/yellow stage and dropped thereafter, particularly in seeds, while *DGAT1* and *DGAT2* were expressed at all stages of development in both pulp and seeds. The relatively low level of *FAD3* expression in the pulp is consistent with the low levels of α-linolenic acid in the pulp oil (Figure 2B), while the reduction in *FAD3* expression in later stages of seed development is consistent with the observed decrease in α-linolenic acid in the orange/red stage (Figure 2A). The high levels of *DGAT1* transcripts at the orange/red stage (Figure 7) is in agreement with the high number of reads obtained for this gene by 454 sequencing (Table 3). *FATB*, which releases palmitic acid from ACP, was also expressed throughout development in seed and pulp tissue, but appeared to be higher in pulp tissue, consistent with the higher proportion of palmitic and palmitoleic acid observed in the pulp oil (Figure 2B).

In *Brassica napus* and Arabidopsis the expression of genes involved in fatty acid biosynthesis, such as *ACC, SAD, FAD2, FAD3*, and *KASI*, is characterized by a bell-shape transcript curve, with moderate levels of expression at the initial stage followed by a gradual increase during the rapid phase of oil accumulation and a subsequent decline toward seed maturation [42], [43]. However, expression of genes involved in TAG assembly pathway, in particular of *DGAT1* and *DGAT3*, remains high throughout stages of seed maturation [43]. The sea buckthorn genes had similar profiles; the relatively high expression of most lipid biosynthesis genes at the green stage reinforced the idea that lipid accumulation was already well underway by that point.

### Comparison of ESTs Corresponding to Fatty Acid Biosynthesis Genes from Sea Buckthorn, Arabidopsis and Flax Seeds

Because Arabidopsis and flax seeds accumulate significant amounts of diverse fatty acids, the available gene expression datasets relevant to fatty acid biosynthesis in these seed systems [44], [45] were used as reference to understand how similar pathways are expressed and regulated in sea buckthorn seeds. We compared the EST counts of key fatty acid biosynthesis genes in the sea buckthorn transcriptome with those in Arabidopsis seeds [44] and developing flax (*Linum usitatissimum* L.) seeds [45]. In addition, we also used normalized microarray values representing expression of Arabidopsis genes [46] to show the general expression trends of these genes. The data represented in Table 4 shows that most components of the fatty acid biosynthesis pathway are represented as ESTs in the sea buckthorn transcriptome, albeit at different levels. The expression levels range from very high for *SAD, DGAT1* and *ACC2* genes to very low levels for *KASI, KASII, KASIII, GPAT1, LPAT4, HAD* and *EAR.* The presence of ESTs in the sea buckthorn seed transcriptome corresponding to genes such as *KASI, KASII, HAD* and *EAR* that peak in expression during the torpedo and cotyledon stage embryos of Arabidopsis and flax, suggests that the regulatory programs involved in the synthesis and deposition of fatty acids are also conserved in sea buckthorn. However, some distinct features in the sea buckthorn seed transcriptome can be seen as considerable enrichment of *DGAT1, FATA* and *FATB* ESTs relative to flax, and much lower representation of *FAD2* and *FAD8* ESTs compared to flax. Interestingly, genes implicated in desaturation and production of linoleic and linolenic acids, such as *FAD 2, 3, 6, 7* and *8* are expressed at similar levels in sea buckthorn seeds, a pattern different from Arabidopsis and flax. This observation is consistent with the 1∶1 ratio of ω-6∶ω-3 fatty acids found in sea buckthorn. Future studies involving expression in heterologous systems may allow us to correlate more definitively the expression patterns of these genes with the oil composition of sea buckthorn seeds.

**Table 4:** Comparison of ESTs corresponding to fatty acid biosynthesis genes from sea buckthorn, flax and Arabidopsis.

Enzyme Symbol	*Arabidopsis* gene ID	Sea buckthorn (mature seed)	Flax(torpedo embryo)	Flax(cotyledon embryo)	Flax(mature embryo)	*Arabidopsis*(developing seed)	*Arabidopsis*(torpedo embryo)	*Arabidopsis*(bent embryo)	*Arabidopsis*(mature embryo)
ACC2	AT1G36180	66	0	0	0	1	2190	2179	2430
KASIII	AT1G62640	8	3	1	0	3	6323	10105	1688
KASI	AT5G46290	4	30	4	0	12	27293	50431	6044
KASII	AT1G74960	5	0	3	0	2	41535	52683	2989
HAD	AT2G22230	3	26	1	0	0	1009	983	975
KAR	AT1G24360	22	17	3	0	8	1940	1770	1617
EAR	AT2G05990	2	26	2	21	0	53147	56170	2580
SAD	AT2G43710	364	14	19	15	12	3023	6907	1372
FAD2	AT3G12120	37	22	119	1125	23	49900	67658	11967
FAD3	AT2G29980	39	7	47	133	10	5131	27776	1123
FAD6	AT4G30950	27	0	1	0	0	2690	2768	1728
FAD7	AT3G11170	41	0	0	1	3	8446	5203	1293
FAD8	AT5G05580	41	8	62	110	0	1525	1223	762
FATA	AT3G25110	17	3	0	0	0	3950	4845	891
FATB	AT1G08510	43	4	0	1	1	4372	4931	3408
GPAT1	AT1G06520	2	0	0	0	2	5138	6008	1703
LPAT4	AT1G75020	4	0	0	0	0	1351	1651	1456
LPP1	AT2G01180	27	0	0	0	0	984	1005	903
LPP2	AT1G15080	27	0	0	0	0	3959	3583	2049
DGAT1	AT2G19450	142	0	0	0	3	9561	7283	14436
DGAT2	AT3G51520	11	1	4	2	0	1435	1510	841
LPCAT	AT1G80950	30	0	0	0	0	889	1039	1233
CPT	AT1G13560	33	0	0	1	3	3340	2154	2926
PDAT	AT5G13640	40	2	1	0	0	693	720	815
**Total reads**	N/A	500392	40412	20514	28856	10738	N/A	N/A	N/A

### Identification of Genes Involved in Isoprenoid Biosynthesis

**Figure 8:**
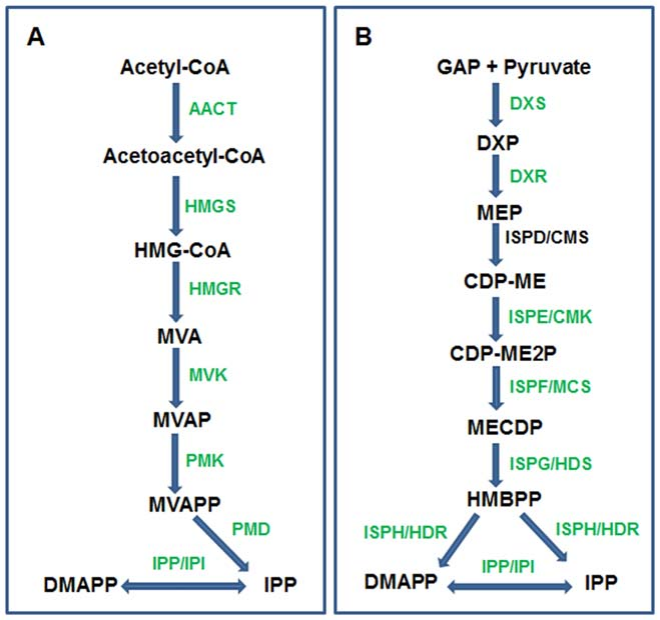
Sea buckthorn sequences associated with isoprenoid biosynthesis by A) cytosolic MVA pathway and B) plastidial MEP pathway. Enzymes for which sea buckthorn sequences have been identified are shown in green. Abbreviations used for intermediates are: 3-hydroxy-3-methyl-glutaryl-CoA (HMG-CoA); mevalonic acid (MVA); mevalonate-5-phosphate (MVAP); MVAPP mevalonate-5-diphosphate (MVAPP); Isopentenyl diphosphate (IPP); Dimethylallyl-diphosphate (DMAPP); d-glyceraldehyde-3-phosphate (GAP); 1-deoxy-D-xylulose-5-phosphate (DXP); 2- C-methyl-D- erythritol-4- phosphate (MEP); 4-(cytidine 5'-diphospho)-2-C-methyl-D-erythritol (CDP-ME); 2-phospho-4-(cytidine 5'C-diphospho) 2-C-methyl-D-erythritol (CDP-ME2P); 2-C- methyl-D- erythritol 2,4-cyclodiphosphate (MECDP); 1-hydroxy-3-methyl-2-(E)-butenyl-4-diphosphate (HMBPP); Abbreviations used for enzymes are: Acetoacetyl-CoA thiolase (AACT); 3-hydroxy-3-methylglutaryl-CoA synthase (HMGS); HMG-CoA reductase (HMGR); mevalonate kinase (MVK); diphosphomevalonate kinase (PMK); diphosphomevalonate decarboxylase (PMD); isopentenyl diphosphate isomerase (IPP/IPI); 1-deoxy-d-xylulose-5-phosphate synthase (DXS); 1-deoxy-d-xylulose-5-phosphate reductoisomerase (DXR); 4-diphosphocytidyl-2-C-methyl-d-erythritol synthase (ISPD/CMS); 4-(cytidine 5′-diphospho-2-C-methyl-d-erythritol kinase (ISPE/CMK); 2-C-methyl-d-erythritol 2,4-cyclodiphosphate synthase (ISPF/MCS); 4-hydroxy-3-methyl but-2-enyl diphosphate synthase (ISPG/HDS); 4-hydroxy-3-methyl but-2-enyl diphosphate reductase (ISPH/HDR).

In plants, isoprenoid compounds are specially abundant and diverse with roles in photosynthesis, respiration, growth regulation and protection against pathogens and herbivores. Isoprenoids are synthesized from the C5 precursor isopentenyl diphosphate (IPP) and its isomer dimethylallyl diphosphate (DMAPP), also called isoprene units [47]. Larger intermediates such as geranyl diphosphate (GPP, C10), farnesyl diphosphate (FPP, C15) and geranylgeranyl diphosphate (GGPP, C20) are derived by sequential addition of isoprene units to DMAPP. These intermediates represent the start points of multiple branches that lead to synthesis of a diverse range of final products such as chlorophyll, carotenoids, plastoquinone, phylloquinone, tocopherol, gibberellin, abscisic acid, phytosterols, brassinosteroids, and phytoalexins, to name a few [48]. Plants use two separate pathways, localized in different cellular compartments, for the synthesis of IPP and DMAPP. The mevalonic acid (MVA) pathway synthesizes IPP and DMAPP in the cytosol (Figure 8A), whereas the methylerythritol 4-phosphate (MEP) pathway synthesizes IPP and DMAPP in plastids (Figure 8B).

Since “isoprenoid biosynthetic process” was also well represented (113 sequences) under the GO term “lipid biosynthetic process” (Table S1), we searched for putative genes involved in the early biosynthetic steps leading to the synthesis of key compounds IPP and DMAPP via the MEP and MVA pathways. With the exception of *AACT, PMK, ISPE* (CMK) and *ISPD* (CMS), sequences corresponding to all other enzymes of the two pathways could be identified under the “isoprenoid biosynthetic process” category (Figure 8, Table S4). Sequences related to *AACT, PMK* and *ISPE* were identified by BLAST analysis of the sea buckthorn dataset in Fiesta 2 with known Arabidopsis sequences. A sequence matching *ISPD* could not be identified in the sea buckthorn seed transcriptome. Analysis of sea buckthorn ESTs related to isoprenoid biosynthesis showed a preponderance of sequences related to *HMGS, HMGR, HDS* (ISPG), *HDR* (ISPH) and *IPP* (IPI). Earlier it was noted that the most abundant ESTs linked to the MEP pathway in Arabidopsis databases, were from genes encoding DXS and HDS, followed by HDR [48]. Since the Arabidopsis ESTs were derived from various tissues and developmental stages, an accurate comparison with sea buckthorn ESTs cannot be made, but the abundance of ESTs encoding HDS and HDR in both plant species suggests that these genes are abundantly expressed. As would be expected, the carotenoid biosynthesis genes [49] were abundantly represented in the seed transcriptome of sea buckthorn (Table S4).

### Identification of Abiotic Stress-related Genes

**Figure 9:**
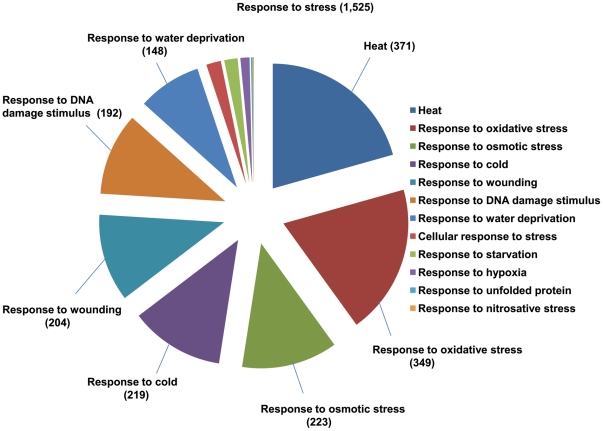
Distribution of Gene Ontology terms within the category “response to stress.”

Since the third most highly represented term within the GO category “biological process”, was “response to stimulus” (2,848 sequences) (Figure 5), we analyzed the distribution of sequences in the subcategory “response to stress” (1525 sequences) (Table S5). Maximum number of sequences fell within the heat stress category (371, 24.3%), followed by oxidative stress (349, 22.8%), osmotic stress (223, 14.6%), cold stress (219, 14.3%), wounding (204, 13.3%), DNA damage (192, 12.5%) and water deprivation (148, 9.7%) (Figure 9). A subset of genes that were recovered as full-length ORFs in the 454 transcriptome, along with their closest Arabidopsis orthologs, is shown in Table 5. Under the heat stress category, several low molecular weight heat shock protein (hsp) genes and Hsp90-related genes were obtained with complete ORFs. Within the dataset represented in Table 5, based on the relative abundance of reads, contigs, and singletons, it can tentatively be inferred that *hsp* genes are the most highly represented set in the mature seed transcriptome. Two factors may account for this: 1) sea buckthorn berry ripening and berry collection in the summer months, which may have triggered accumulation of hsps, and 2) the established expression and functions of various hsps during embryo development and maturation, and seed germination in various plant species [50]–[52]. The *AtRZ-1A* in Arabidopsis affects seed germination and seedling growth under low temperature [53]. The relatively high number of contigs and singletons corresponding to *AtRZ-1* family members in the sea buckthorn seed transcriptome described here suggests a possible developmental and/or stress-protective role in seed germination for these genes. The Arabidopsis *PDX1* and *RCI2A* genes have roles in seed development and stress tolerance, with the former involved also in vitamin B6 biosynthesis [54], and the latter responding to environmental stimuli, such as low temperature, dehydration, salt stress, and abscisic acid (ABA) [55]. The late embryogenesis abundant (LEA) proteins, including LEA14, accumulate in the embryo during seed maturation and are associated with dehydration in seeds [56]; however, AtLEA5 (also called SAG21) is constitutively expressed in roots and reproductive organs but not in seeds, and has a specific role in protection against oxidative stress [57].

Early response to dehydration (ERD) proteins play roles in seed development and germination as well as protection of plants from stresses such as cold and dehydration [58], while DREB2 proteins are transcription factors that regulate the expression of stress-responsive genes [59]. The multiprotein bridging factor 1 (MBF1) gene family encodes transcriptional co-activators, which are suggested to function as regulatory components of cross-talk between ethylene, ABA and stress signal pathways [60]. Histone deacetylase 2 (HD2) proteins are plant-specific histone deacetylases (HDAC) that do not share sequence similarities with other known HDACs. *AtHD2*s are highly expressed in embryos and also strongly induced during the process of somatic embryogenesis [61]. Specifically, AtHD2C can modulate ABA and stress responses [62]. By comparison to other stress categories, less is known about the expression patterns and functions of the genes under the DNA repair category in Arabidopsis. In conclusion, numerous stress-related genes with key roles in seed development and maturation were identified in the sea buckthorn transcriptome. Which of these are important players in the stress tolerance potential of sea buckthorn will only be revealed when more is learnt about the expression and functions of the gene complements in this plant species.

**Table 5:** Full-length genes related to abiotic stress response in sea buckthorn seed transcriptome, along with nucleotide identities between Arabidopsis genes and sea buckthorn sequences.

Stress	Protein name	Description	*Arabidopsis* gene ID	Sea buckthorn contigs+singletons	Reads/gene	Contig with full-length ORF	Contig Length (nt)	Global gene identity (%)	Local gene identity (%)
Heat	HSP81-1/HSP90.1	Heat shock protein90.1, ATP binding	AT5G52640	55	824	CL1Contig4975	2526	65.5	78.2
	HSP81-2/HSP90.2	Heat shock protein 81-2, ATP binding	AT5G56030	55	824	CL1Contig3994	2339	74.2	82.8
	HSP81-3	HSP81-3; ATP binding	AT5G56010	55	824	CL1Contig3994	2339	74.2	82.7
	sHSP18.2	Heat shock protein 18.2	AT5G59720	155	3263	CL1Contig1554	748	44.8	67.8
	sHSP17.8	17.8 kDa class I heat shock protein	AT1G07400	146	3245	CL1Contig4813	884	36.8	67.3
	sHSP17.4	17.4 kDa class III heat shock protein	AT1G54050	32	2730	CL1Contig4535	1466	20.0	58.6
	sHSP26.5	26.5 kDa class I small heat shock protein-like	AT1G52560	59	1147	CL2Contig8	758	59.7	61.2
Cold	ATRZ-1A	ATRZ-1A; RNA binding	AT3G26420	128	2949	CL1Contig2740	909	47.0	63.2
Osmotic	SHP	hydrophobic, low temperature and salt responsive protein	AT2G38905	17	110	CL5241Contig1	291	42.1	73.8
	PDX1	Pyridoxine biosynthesis 1.1, protein heterodimerization	AT5G01410	11	158	CL302Contig1	1339	53.1	76.1
	RCI2A	Rare-cold-inducible 2A	AT3G05880	17	110	CL5241Contig2	364	28.8	68.2
Water deprivation	LEA14	Late embryogenesis abundant 14	AT1G01470	9	344	CL58Contig1	626	48.0	62.6
	ERD15	Early resonsive to dehydration 15, protein binding	AT2G41430	4	143	CL396Contig1	1131	23.1	42.2
	DREB2B	DRE/CRT-binding Protein 2B, DNA binding	AT3G11020	62	1857	CL1891Contig1	1258	36.1	46.5
	MBF1C	Multiprotein bridging factor 1C, DNA binding	AT3G24500	14	440	CL1Contig4366	815	37.5	68.6
	SAG21	Senescence associated gene 21	AT4G02380	5	314	CL1Contig1230	655	28.2	62.4
	HD2C	Histone deacetylase 2C/nucleic acid binding	AT5G03740	4	259	CL1308Contig2	1255	40.1	55.6
DNA repair	CID7	ATP binding/damaged DNA binding/protein binding	AT2G26280	15	139	CL250Contig5	2168	53.0	66.3
	DNA Binding	DNA binding/hydrolase, acting on ester bonds	AT3G52905	3	18	CL2978Contig1	1026	33.8	62.0
	RPA2	DNA binding/nuclease	AT2G24490	2	12	CL5263Contig1	1272	37.0	54.6

In conclusion, this study provides a comprehensive account of fatty acid composition in sea buckthorn fruits collected from Canadian-grown cultivars. Our results indicate that the seed oil contains ω-3 and ω-6 fatty acids that play an important role in human health at high levels and in a desired ratio of 1∶1, and the pulp oil contains high levels of ω-7 fatty acid, which promotes skin health and has commercial applications. In addition, this study provides the first transcriptome data for sea buckthorn, which has identified in this plant most known genes related to fatty acid biosynthesis. Both fatty acid and gene expression profiles in developing seeds and fruits of sea buckthorn indicate that the early stages of fruit development are optimal for oil quality from a nutritional standpoint. Such correlating information at the genetic and chemical levels will lead to superior product development and breeding tools that will help select germplasm with optimal levels of bioactive compounds.

## Methods

### Plant Material

Sea buckthorn cultivars RC-4 and FR-14 were selected from a seedling population of Indian-Summer (*H. rhamnoides* ssp. *mongolica*) growing in shelterbelts and wildlife plantings near Estevan, Saskatchewan, Canada. Sea buckthorn cultivars E6590 and Harvest Moon were selected from an open-pollinated seedling population of *H. rhamnoides* ssp. *mongolica* growing at the Agriculture and Agri-Food Canada, Agroforestry Development Centre near Indian Head, Saskatchewan. Fully ripe fruits (500 g) were harvested from these four cultivars in the field trial in August 2007, immediately frozen in liquid nitrogen and stored at −80°C until further use. The RC-4 seeds were isolated from frozen fruits and used for extracting total RNA for cDNA library construction. Lipid analysis was performed on all four cultivars (RC-4, E6590, Harvest Moon and FR-14). The whole berries of RC-4 cultivar at different developmental stages were harvested in the field trial during August to October 2009. The four developmental stages described by fruit color green (G: 6 August); green/yellow (G/Y: 17 August); yellow/orange (Y/O: 31 August) and orange/red (O/R: 22 October) were used for lipid analysis, and gene expression profiling by RT-PCR.

### Lipid Extraction

The oil from sea buckthorn fruit tissues was extracted according to the method of Hara and Radin [63]. Briefly, isopropanol (2 mL) was added to a known weight of seed, pulp or whole berry and the mixture was heated at 85°C for 10 min. Samples were then homogenized for 1 min after adding 2 mL hexane and 4 mL hexane:isopropanol (3∶2 v/v). After homogenizing, the total volume was brought up to 12 mL using 3∶2 hexane:isopropanol. To this mixture, aqueous sodium sulphate (4 mL of 3.3% w/v) was added to induce phase separation. The organic phase was removed and the aqueous phase re-extracted with 8 mL 7∶2 hexane:isopropanol. The combined lipid extracts were evaporated under nitrogen, then resuspended in hexane (10 mg/mL for seed extracts, 100 mg/ml for pulp and berry extracts) and stored at –20°C under nitrogen until further analysis.

### GC-MS Analysis of Fatty Acid Analysis in Total Lipid Extracts of Whole Berries, Pulp and Seeds

Methanolic HCl was prepared by the gradual addition of 20 mL acetyl chloride to 100 mL cold methanol. Fatty acid methyl esters (FAMEs) were prepared from total lipid extracts of sea buckthorn fruit tissues by adding 1mL of methanolic HCl to 1 mg of total lipid and incubating for 1 h at 80°C. The methylation was quenched by the addition of 1 mL 0.9% aqueous sodium chloride, and the FAMEs were extracted twice with 2 mL hexane. The resulting FAMEs extract was evaporated under nitrogen and resuspended in iso-octane for GC/MS analysis.

GC/MS analysis of FAMEs was performed on an Agilent 6890N gas chromatograph with an Agilent 5975B Inert XL mass selective detector. Chromatographic separation was achieved using a DB-23 capillary column (J&W Scientific, Folsom CA; 30 m×250 µm×0.25 µm) with the following temperature program: initial temperature 90°C, raised at 10°C/min to 165°C, held for 5 min, then raised at 3°C/min to a final temperature of 230°C. The inlet was operated in splitless mode at a temperature of 290°C, with helium as the carrier gas at constant flow of 1.2mL/min. The transfer line temperature was 250°C, and the MS ion source and quadrupole were set to 230°C and 150°C, respectively. MS detection was carried out in electron impact (EI) ionization mode, scanning all masses from 30–350 amu. Peaks were identified based on mass spectral comparison with the NIST05 MS library in combination with retention time matching to external FAMEs standards (Nu-Chek Prep, Elysian, MN; Standard #421A, with 100 µg/mL 18∶1*cis*11 FAME added).

### Total RNA Extraction from Sea Buckthorn Seed and Pulp

RNA was extracted from sea buckthorn seeds [64]. Briefly, 0.5–1.0 g of seeds was ground in liquid nitrogen using sterile pestle and mortar. To the seed powder, 2 mL of basic RNA extraction buffer (100 mM Tris-HCl, pH 9.0, 20 mM EDTA, 4% (w/v) sarkosyl, 200 mM NaCl, 16 mM DTT, and 16 mM mercaptobenzothiazol) containing 5% (w/v) BSA and 10 mg/mL heparin were added followed by the addition of 1.5 g of hydrated PVPP. The mixture was ground to a homogenate for 1 min and then 6.5 mL of basic RNA extraction buffer (no additions) were added. After homogenizing for 1 min, 200 µL proteinase K (10 mg/mL stock) were added, followed by another 1 min of grinding. The mixture was transferred to a 24 mL polypropylene screw-cap tube and incubated at 37°C with gentle shaking for 20 min before centrifugation at 14,000×*g* in Sorvall* *RC−3 SS34 rotor for 10 min at 4°C. The supernatant was transferred to a new tube and extracted twice with an equal volume of phenol and once with phenol:chloroform:isoamyl alcohol (25∶24:1). Centrifugation for each extraction was conducted at 11,000×*g* for 15 min at 20°C. The final upper phase was transferred to a 15 mL sterile polypropylene tube to which 1/3 volume of 8 M LiCl was added and the mixture was incubated at 4°C overnight. Following centrifugation at 11,953×*g* in the SS34 rotor for 20 min at 4°C, the pellet was recovered and washed twice with 2 M LiCl. The pellet was dissolved in 2 mL of RNase-free water, followed by ethanol precipitation. The RNA pellet was recovered by centrifugation at 11,953×*g* for 30 min at 4°C and rinsed with 70% ethanol in RNase-free water. The pellet was dried and dissolved in 50 µL of RNase-free water. Further purification was carried out using the RNeasy Plant Mini kit (Qiagen Inc. Mississauga, Ontario, Canada) coupled with on-column DNase digestion. RNA quality was assessed using Agilent Technologies 2100 Bioanalyzer (Agilent Technologies). Good quality RNA could be obtained from seeds, but not pulp, using this method.

To isolate RNA from seed and pulp tissues of berries at four developmental stages, the protocol described [65] was employed with minor modifications. Briefly, 10 mL 90% acetone was added to ground 0.7–1.0 g powder, and the mixture was centrifuged at 14,000×*g* in the SS34 rotor for 10 min at 4°C. The pellet was washed with 80% acetone by centrifugation at 14,000×*g* for 10 min at 4°C. To the dried pellet, 10 mL buffer (0.2 M Tris-HCL pH 8.0, 1.4 M NaCl, 1% CTAB, 0.02 M EDTA pH 8.0, 1% sodium deoxycholate, 2% β- mercaptoethanol) pre-warmed at 60°C was added, followed by incubation at 60°C for 30 min. An equal amount of chloroform was added and the mixture was incubated for 15 min at 60°C and then centrifuged at 14,000×*g* for 10 min. This extraction was repeated with the aqueous phase and the RNA was precipitated overnight with 3 M LiCl at 20°C. The RNA pellet was collected by centrifugation at 17,000×*g* for 30 min, dissolved in 0.5 mL of RNase-free water and extracted once with phenol, once with phenol:chloroform (1∶1) and once with chloroform, with each extraction for 5 min at room temperature. Finally, the RNA was precipitated with NaOAc (pH 5.2) and absolute ethanol at −70°C. The pellet was washed with 70% ethanol, dried and dissolved in 100 µL of RNase-free water and stored at −70°C until further use.

### Construction of cDNA Library

Poly(A) mRNA was isolated from total RNA using the Oligotex mRNA Midi Kit (Qiagen Inc.) and the quality was assessed using the 2100 Bioanalyzer. Samples with high RNA integrity numbers (RIN) were selected for further processing. Double-stranded (ds)-cDNA was synthesized from mRNA using the cDNA Synthesis System (Roche, Mississauga, Ontario, Canada). Ten individual reactions were performed for ds-cDNA synthesis. First-strand cDNA synthesis was done using 1 µg of mRNA and random hexamer primers instead of oligo (dT). The Second-strand cDNA synthesis was performed according to the manufacturer's instructions. The ds-cDNA was cleaned using the column-based High Pure PCR Cleanup Micro kit (Roche, Mississauga, Ontario, Canada). cDNAs were sheared by nebulization to yield fragments approximately 500 bp in length. Standard procedures for end polishing and purification, adaptor ligation, isolation and estimation of ssDNA concentration, emulsion PCR with library beads and final loading on the 454 plates were performed according to supplier’s instructions (Roche, Mississauga, Ontario, Canada ). The sequencing was conducted at Plant Biotechnology Institute (PBI) of the National Research Council of Canada (Saskatoon, Canada) using an FLX model 454 DNA Sequencer (Roche, Mississauga, Ontario, Canada).

### Transcript Assembly and Gene Ontology Annotation of Sea Buckthorn Unigenes

The 454 reads (500,392) were assembled and annotated using two sequence data management (SDM) and analysis platforms, Fiesta 2 and GenomeQuest. The trimmed 454 read sequences were assembled in Fiesta 2 using TGICL software from TIGR (www.ncbi.nlm.nih.gov/pubmed/12651724). Overlapping reads were assembled into contigs, and sequences that appeared only once in the ESTs were classified as singletons. The sequences were archived in the Fiesta 2 software package (http://bioinfo.pbi.nrc.ca/napgen.beta//login.html) at PBI. The unigenes were annotated against both Arabidopsis and Uniprot Plants using BLASTX (E-value cutoff 10–6). Gene Ontology (GO) terms were used to identify possible functional classifications of the unigenes via assignment of Arabidopsis gene identifiers with the strongest BLASTX alignments to the corresponding sea buckthorn EST.

On the GenomeQuest SDM platform, raw sequence files (SFF format) were modified based on the adaptor trimming information and the modified files were processed using the Roche Newbler assembly program. Due to a limit of 300,000 sequences that can be processed at one time by this software in GenomeQuest, the 500,392 raw reads were split into two buckets of 250,196 sequences in FASTA for assembly by Newbler. The unigenes were annotated with their best hits in public databases by running Rapid Annotation Process (RAP) against PLN (plant, fungal, and algal sequences) in GenBank to find distribution of sea buckthorn sequences against other organism sequences.

### Computational Metabolic Network Prediction, and Identification of Genes Involved in Fatty Acid Biosynthesis and Abiotic Stress

MetaCyc (MetaCyc.org) is a non-redundant reference database that contains experimentally verified metabolic pathway and enzyme information. The largest category of pathways in MetaCyc is biosynthesis, with 530 base pathways [66]. The “biosynthesis” category consists of secondary metabolites biosynthesis (198 pathways), amino acids biosynthesis (91), cofactors, prosthetic groups and electron carriers biosynthesis (83) and fatty acids and lipids biosynthesis (51). We used MetaCyc in conjunction with the PathoLogic component of the Pathway Tools software (http://www.ncbi.nlm.nih.gov/pubmed/12169551) to computationally predict the metabolic network of sea buckthorn seeds.

Genes related to fatty acid and TAG biosynthesis were identified using PlantCyc (http://pmn.plantcyc.org/), a comprehensive plant metabolic pathway database that provides access to shared and unique metabolic pathways present in over 300 plant species, including Arabidopsis. The Arabidopsis database [the AraCyc Pathways Database (www.arabidopsis.org/tools/aracyc/)] was searched for specific enzymes involved in each step of the fatty acid biosynthesis pathway. The Arabidopsis enzyme sequences were used to query the sea buckthorn dataset in Fiesta 2 (E-value cutoff, 1e-5) and the total number of hits (number of contigs+singletons, reads per gene), and their lengths were recorded. Nucleotide sequence comparisons between Arabidopsis coding sequences and sea buckthorn sequences were done by local and global alignment programs (http://www.ebi.ac.uk/Tools/psa). Sequences from sea buckthorn seed were searched in the GO subcategory “response to stress” arising from the GO category “response to stimulus”, and analysed for full-length coding sequences. The total number of hits, reads per gene and % identities were calculated as described before.

### RT-PCR Analysis

Total RNA was isolated from frozen seed and pulp tissue of RC-4 to represent two independent biological replicates. cDNA synthesis was performed with 1 µg of total RNA using QuantiTect Reverse Transcription kit (Qiagen Inc. Mississauga, Ontario, Canada) according to manufacturer’s instructions. Semi-quantitative RT-PCR was performed with EST-specific primers (Table S6), designed using Primer3 software. The amplification conditions were 94°C for 4 min for initial denaturation, followed by 30 cycles of denaturation (30 s at 94°C), annealing (45 s at 56°C) and extension (1 min at 72°C) with final extension for 4 min at 72°C. The *ubiquitin5* gene was used as internal control with 25 PCR cycles.

The sequences have been submitted to NCBI’s Short Read Archive (SRA) with Sample accession number, SRS290823.1.

## Supporting Information

Figure S1 **Sea buckthorn fruits at four developmental stages.** Fruits from RC-4 cultivar were harvested in the field trial during August to October 2009.
(TIF)

Figure S2 **Distribution of Gene Ontology terms within the category “metabolic process.”**
(PDF)

Table S1 **GO terms for biosynthetic process, lipid biosynthetic process and isoprenoid biosynthetic process.**
(DOCX)

Table S2 **A complete list of contigs and singletons annotated with GO category ‘Fatty acid biosynthetic process’ and ‘Glycerolipid biosynthetic process’.**
(XLSX)

Table S3 **A detailed version of** **Table 3** **showing all contigs and singletons related to fatty acid biosynthesis genes.**
(XLSX)

Table S4 **A complete list of contigs and singletons annotated with GO category ‘Isoprenoid biosynthetic process’. Green colour represents enzymes shown in** **Figure 8** **.**
(XLSX)

Table S5 **List of genes annotated with GO category ‘Abiotic stress’, the most closely related sea buckthorn contig, and the total number of unigenes related to a stress gene.**
(XLSX)

Table S6 **Sequences of primers used in RT-PCR analysis.**
(DOCX)
