# Supplementary figures and images for: Fatty Acid Composition of Developing Sea Buckthorn (Hippophae rhamnoides L.) Berry and the Transcriptome of the Mature Seed
Source: PLoS One. 2012 Apr 27;7(4):e34099. doi: 10.1371/journal.pone.0034099 (PMC3338740; doi:10.1371/journal.pone.0034099)

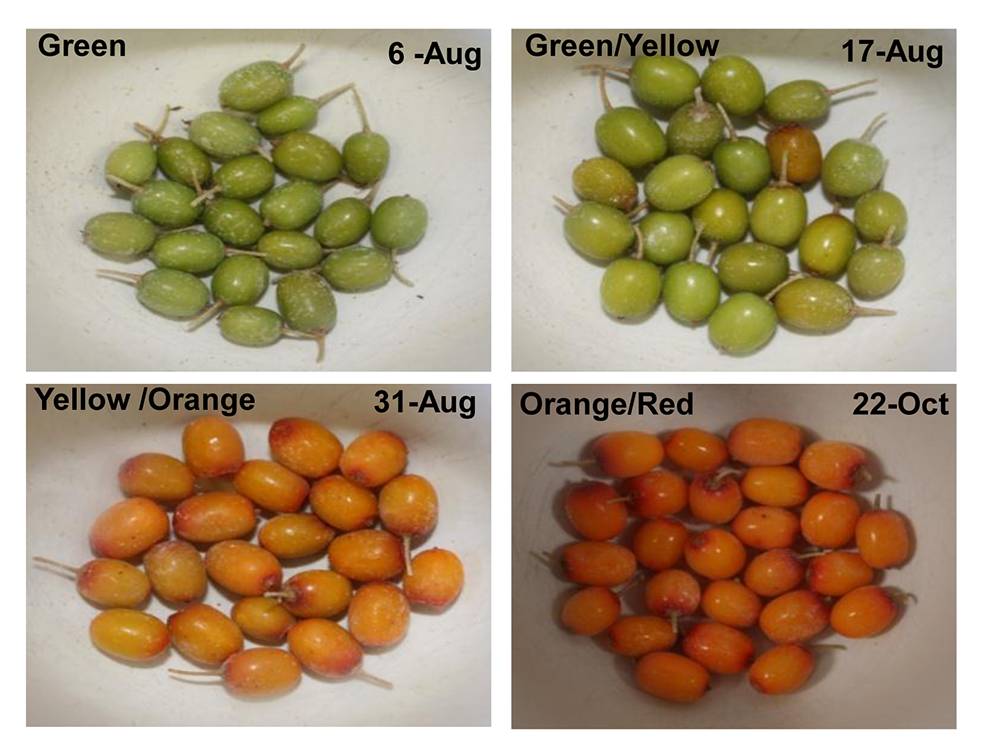

Supplement: Figure S1 — Sea buckthorn fruits at four developmental stages. Fruits from RC-4 cultivar were harvested in the field trial during August to October 2009. (TIF) [file pone.0034099.s001.tif]

# Metabolic process (13,010)

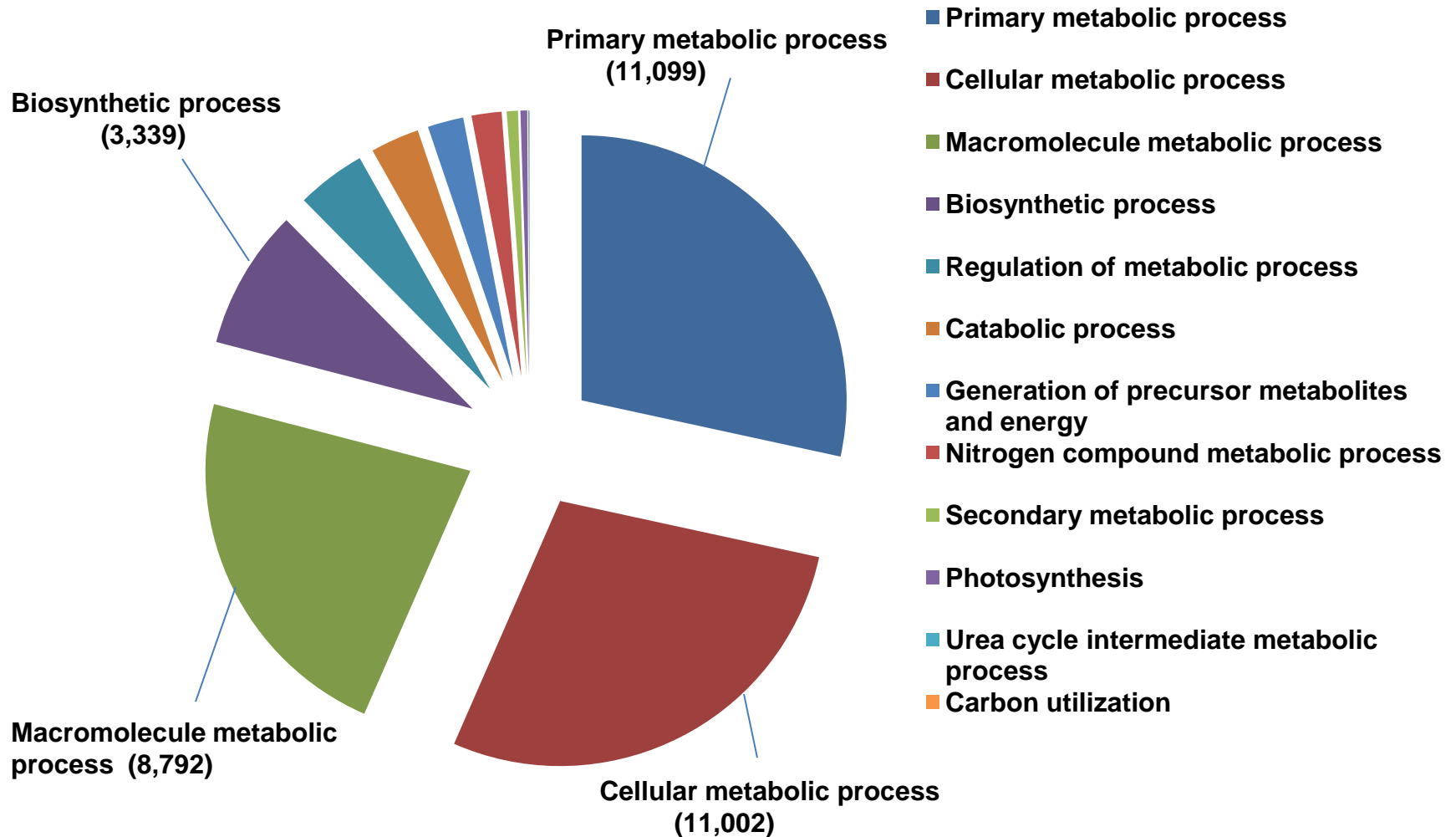

Supplement: Figure S2 — Distribution of Gene Ontology terms within the category “metabolic process.” (PDF) [file pone.0034099.s002.pdf]
