# Supplementary material for: Fatty Acid Composition of Developing Sea Buckthorn (Hippophae rhamnoides L.) Berry and the Transcriptome of the Mature Seed
Source: PLoS One. 2012 Apr 27;7(4):e34099. doi: 10.1371/journal.pone.0034099 (PMC3338740; doi:10.1371/journal.pone.0034099)
Supplement: Table S1 — GO terms for biosynthetic process, lipid biosynthetic process and isoprenoid biosynthetic process. (DOCX) [file pone.0034099.s003.docx]

**Table S1: GO terms for biosynthetic process, lipid biosynthetic process and isoprenoid biosynthetic process.**

| **Biosynthetic process (GO: 0009058) 3339 sequences (3.75%)** | |
| --- | --- |
| Macromolecule biosynthetic process | 1964 sequences (2.20%) |
| Cellular biosynthetic process | 1222 sequences (1.37%) |
| **Lipid biosynthetic process** | **434 sequences (0.49%)** |
| Regulation of biosynthetic process | 69 sequences (0.08%) |
| Cuticle hydrocarbon biosynthetic process | 8 sequences (0.01%) |
| Peptidyl-diphthamide biosynthetic process  from peptidyl-histidine | 2 sequences (0.00%) |
| **Lipid biosynthetic process (GO: 0008610) 434 sequences (0.49%)** | |
|  |  |
| **Fatty acid biosynthetic process** | **153 sequences (0.17%)** |
| **Isoprenoid biosynthetic process** | **113 sequences (0.13%)** |
| Membrane lipid biosynthetic process | 112 sequences (0.13%) |
| Steroid biosynthetic process | 25 sequences (0.03%) |
| Glycerolipid biosynthetic process | 21 sequences (0.02%) |
| Neutral lipid biosynthetic process | 21 sequences (0.02%) |
| Sulfolipid biosynthetic process | 5 sequences (0.01%) |
| Lipopolysaccharide biosynthetic process | 4 sequences (0.00%) |
| Regulation of lipid biosynthetic process | 4 sequences (0.00%) |
|  |  |
| **Isoprenoid biosynthetic process (GO: 0008299) 113 sequences (0.13%)** | |
| Terpenoid biosynthetic process | 53 sequences (0.06%) |
| Isopentenyl diphosphate biosynthetic process | 16 sequences (0.02%) |
| Prenol biosynthetic process | 2 sequences (0.00%) |
| Others | 42 sequences (0.04%) |

**GO term > biological process >metabolic process >biosynthetic process > lipid biosynthetic process > fatty acid biosynthetic process /** **Isoprenoid biosynthetic process**
