# Supplementary material for: Fatty Acid Composition of Developing Sea Buckthorn (Hippophae rhamnoides L.) Berry and the Transcriptome of the Mature Seed
Source: PLoS One. 2012 Apr 27;7(4):e34099. doi: 10.1371/journal.pone.0034099 (PMC3338740; doi:10.1371/journal.pone.0034099)
Supplement: Table S6 — Sequences of primers used in RT-PCR analysis. (DOCX) [file pone.0034099.s008.docx]

**Table S6. Sequences of primers used in RT-PCR analysis**

| **Primer** | **Forward (5’-3’)** | **Reverse (5’-3’)** |
| --- | --- | --- |
| SbtACC2 | AGGATACCAGCCAACACAAA | ACTTCTTCAGCCACACCAAT |
| SbtKASIII | TTTGGAGATGCTGCTGGTG | CGTATGCCATAAAGTTCAGGACT |
| SbtKAR | GAGTCTATGATTAAAACTGTGGTTGA | TTTACATTACCATTCCCCCATC |
| SBtFATB | CCCAGCTTTGGCTATTGTGT | CATCCCGAACATCATCACTG |
| SbtEAR | CCCGCTTTGAACATTTTTGA | TCCAGCAGAGGTTTGGTGAC |
| SbtFAD8 | ATTGTTTTCCCCCAATGAAA | CCAAATGTCTGATCAAAAGTGG |
| SbtFAD7 | CCCCCAATGAAAGGAAACA | TGTCTGATCAAAAGTGGAATCCA |
| SbtFAD3 | GAATAGCTTTGTTGGGCATATTTT | AACTCCATAATCTCTATCAATTG |
| SbtFAD2 | GCCACTTTGATCCTCATGG | TCCCCAAACACAGATAGCATAATC |
| SbtDGAT2 | GCGAGAAGAGGATTTGTTCG | TCAGAGAATTTGCAATTGGAGATC |
| SBTDGAT1 | CCACAACCACAATTTTGTATCC | TTGATCAGTTGACGGAAAACC |
| SbtUBQ5 | AAGGCAAAGATACAAGACAAGGA | TGAGTCCACACTTACCACAATAG |
